# Efficacy of sofpironium bromide gel on clozapine-induced hypersalivation in patients with treatment-resistant schizophrenia: double-blind, controlled crossover study

**DOI:** 10.1192/bjo.2022.630

**Published:** 2023-01-13

**Authors:** Yuhei Amano, Jun Mazda, Koichi Amano, Kazutaka Ohi, Toshiki Shioiri

**Affiliations:** Anzunokai Kakamigahara Hospital, Kakamigahara, Japan; and Department of Psychiatry, Gifu University Graduate School of Medicine, Gifu, Japan; Anzunokai Kakamigahara Hospital, Kakamigahara, Japan; Department of Psychiatry, Gifu University Graduate School of Medicine, Gifu, Japan

**Keywords:** Hypersalivation, clozapine, schizophrenia, sofpironium bromide, anticholinergic gel

## Abstract

**Background:**

Hypersalivation is a major side-effect of clozapine in patients with treatment-resistant schizophrenia.

**Aims:**

We investigated the efficacy of topical anticholinergic formulation sofpironium bromide gel for improving hypersalivation in patients with treatment-resistant schizophrenia receiving clozapine.

**Method:**

A double-blind, controlled crossover study was conducted with sofpironium bromide gel and a placebo gel to treat clozapine-induced hypersalivation in 16 patients with treatment-resistant schizophrenia. Patients were randomly divided between groups A and B (each *n* = 8). Group A was treated with sofpironium bromide gel for 6 weeks, followed by a 2-week washout period and 6 weeks of placebo gel, after which they were observed for another 2 weeks. In contrast, group B was treated with placebo gel for 6 weeks, followed by a 2-week washout period, 6 weeks of sofpironium bromide gel and a 2-week observation period. One-minute saliva volume, objective salivation ratings (Drooling Severity and Frequency Scale and Nocturnal Hypersalivation Rating Scale) and subjective salivation ratings (Visual Analogue Scale) were assessed every 2 weeks.

**Results:**

All patients completed the trials. Three patients reported mild, spontaneously resolved skin itching. Compared with baseline values, the 1-min saliva volumes of both groups were significantly decreased by approximately 30% at the second week of sofpironium bromide gel treatment (*P* < 0.001), and significantly decreased by >40% at the fourth and sixth weeks of treatment (*P* < 0.001). The effects were maintained for over 2 weeks even after the treatment was discontinued.

**Conclusions:**

We suggest that sofpironium bromide gel is effective in treating clozapine-induced hypersalivation in patients with treatment-resistant schizophrenia.

Clozapine, one of the most established antipsychotics for treatment-resistant schizophrenia, is widely prescribed at present.^1^ However, its side-effects, such as agranulocytosis, impaired glucose tolerance and constipation, can be difficult to manage.^1–3^ Another side-effect of clozapine is hypersalivation, which negatively affects patients’ quality of life and can cause aspiration pneumonia.^4,5^ Hypersalivation occurs in 31.0–97.4% of patients treated with clozapine.^6^ It occurs more often at night than during the day, but occurs frequently even during the day or at levels that negatively affect quality of life in 20.4% of patients.^5^ The severity of hypersalivation depends on the dose and blood concentration of clozapine.^7,8^ Hypersalivation is not a common side-effect for antipsychotics overall, since some antipsychotics often induce dry mouth as a side-effect. A significant increase in saliva volume can be observed 3 weeks after the start of clozapine administration,^9^ and occurs at a significantly higher rate with clozapine than with olanzapine, which has a similar drug profile,^10^ suggesting that it is a clozapine-specific side-effect.

The pharmacological basis of clozapine-induced hypersalivation is unknown, but various mechanisms, such as activation of muscarinic M4 receptors, antagonism of α2-adrenergic receptors and inhibition of the swallowing reflex, may be involved in clozapine-induced hypersalivation.^6,11,12^ Because clozapine and its major metabolite, *N*-desmethylclozapine, have antagonistic and agonistic effects on various muscarinic receptors,^13,14^ it is assumed that the action on muscarinic receptors is the primary cause.^15^ In Sjögren's syndrome, a disease associated with decreased saliva, cholinergic drugs that act on muscarinic M3 receptors of the salivary gland are used as the standard treatment to increase salivation.^16,17^ Thus, cholinergic drugs are understood to increase salivation, whereas anticholinergic drugs are understood to have the opposite effect on the salivary gland. There is currently no approved drug to treat clozapine-induced hypersalivation. Although oral administration or injection of anticholinergic drugs such as scopolamine,^15^ trihexyphenidyl,^18^ propantheline,^10^ pirenzepine,^19^ diphenhydramine,^10^ metoclopramide,^20^ clonidine,^9^ amisulpride^21^ and *Clostridium botulinum* toxin^22–24^ have been reported to be effective, there are side-effects, such as a severe decrease in gastrointestinal motility, urinary retention^25,26^ and injection pain; additionally, a meta-analysis has indicated an increase in constipation.^10^ Therefore, anticholinergics should be used with caution, and further evidence is needed before they can be established as a treatment for clozapine-induced hypersalivation.^27^ Glycopyrrolate is an anticholinergic agent with a quaternary ammonium structure, limiting its passage across the blood–brain barrier, thereby greatly reducing the risk for central anticholinergic adverse effects. Recently, a randomised, crossover, double-blind, placebo-controlled trial has demonstrated the efficacy and safety of glycopyrrolate in patients with clozapine-associated sialorrhea (*N* = 32; the difference in the efficacy between drug and placebo groups was over 35%).^4^

## Sofpironium bromide gel

Patients with intractable neurological diseases, such as amyotrophic lateral sclerosis (ALS) and Parkinson's disease, experience hypersalivation similar to patients receiving clozapine therapy.^28^ An external anticholinergic preparation, 5% scopolamine ointment, has been used to treat them. Although this drug is not commercially available, the use of such a drug is recommended to treat drooling in Japanese guidelines for ALS (*N* = 30 patients; the difference in the efficacy between drug and placebo groups was over approximately 30%).^29,30^ Therefore, in 2020, insurance coverage was approved in Japan for sofpironium bromide gel (5% ECCLOCK® gel), an external preparation of an anticholinergic drug with M1–5 muscarinic receptor antagonistic action against primary axillary hyperhidrosis.^23^ In the Japanese phase 3 trial (patients who received sofpironium bromide gel (141 patients) or placebo (140 patients)), in addition to the efficacy (the difference was 17.5% between two groups), the incidence of constipation caused by sofpironium bromide gel was extremely low (0.7%). The main adverse events were limited to localised skin reactions such as dermatitis and erythema, making this preparation extremely useful in clinical settings.^23^ As a derivative of glycopyrronium, sofpironium bromide consists of a chemically modified structure that allows the drug to undergo rapid hydrolytic deactivation, and thus minimise the significant side-effects associated with traditional anticholinergic drugs. Furthermore, the retrometabolic drug design of topical sofpironium bromide presents distinct advantages by limiting systemic absorption and therefore development of anticholinergic adverse events. Therefore, local external administration of anticholinergic drugs can reduce systemic side-effects compared with oral administration or injection of anticholinergic drugs.

In this study, we aimed to test the efficacy of sofpironium bromide gel^23^ for improving hypersalivation by applying the gel to the external skin over the parotid and submandibular glands in patients with treatment-resistant schizophrenia receiving clozapine therapy. Evidence of its efficacy should add a new therapeutic option for clozapine-induced hypersalivation. Because it is a topical formulation that acts locally, it uses a lower dose than the conventional oral administration of anticholinergic drugs, which is likely to reduce systemic side-effects.

## Method

### Participants

We selected in-patients or out-patients with treatment-resistant schizophrenia who were: aged ≥20 years; diagnosed with schizophrenia according to the DSM-5; receiving clozapine therapy for ≥8 weeks and experiencing hypersalivation, with total severity and frequency scores on the Drooling Severity and Frequency Scale (DSFS)^24,31^ indicating moderate or severe hypersalivation (i.e. five or more points). The exclusion criteria were as follows: clearly worsening psychiatric symptoms in the past 4 weeks; worsening physical condition in the past 4 weeks; use of oral anticholinergics; angle-closure glaucoma, dysuria owing to prostatic hyperplasia or hypersensitivity to sofpironium bromide gel components; or other factors, such as pregnancy and severe dermatitis, deemed to be disqualifying by a physician.

Among the 70 patients (27 men and 43 women) with treatment-resistant schizophrenia, 19 (27%) had hypersalivation, with total DSFS scores for severity and frequency of five or more points, which was consistent with frequencies in previous studies of patients with treatment-resistant schizophrenia who received clozapine.^6,24^ Of 19 patients, written informed consent was obtained from 16 participants (seven men and nine women) after the procedures had been thoroughly explained. The authors assert that all procedures contributing to this work comply with the ethical standards of the relevant national and institutional committees on human experimentation and with the Helsinki Declaration of 1975, as revised in 2008. This study was approved by the Nagoya City University Clinical Research Review Committee (approval number CRB4200003) and was published in the Japan Registry of Clinical Trials (identifier jRCT1041210028). We recruited and registered our participants from 20 July to 26 August 2021.

### Procedure

A prior sample size calculation was performed with an expected clinically improvement (mean difference ± s.d. 0.30 ± 0.20) in saliva volume between before and after sofpironium bromide gel treatment during the double-blind phase, according to our preliminary examination of the sofpironium bromide gel intervention in healthy medical staff and previous studies on glycopyrrolate in patients with clozapine-associated sialorrhea,^4^ using an alpha of 0.05 with 80% power. A sample size (*N* ≥ 10) was required by the sample size estimation.

**Fig. 1:**
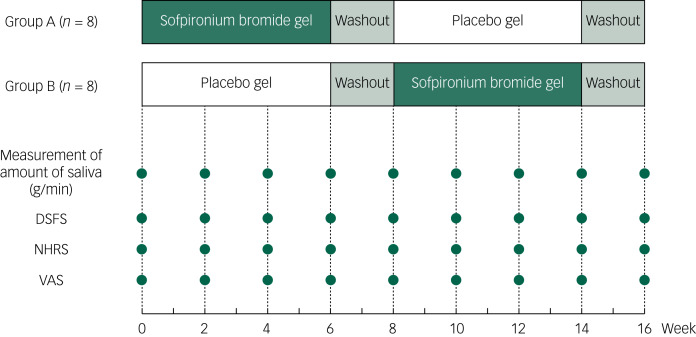
Study protocol. Assessments of saliva volume (g/min); DSFS, NHRS and VAS measurement timepoints are indicated by filled circles. DSFS, Drooling Severity and Frequency Scale; NHRS, Nocturnal Hypersalivation Rating Scale; VAS, Visual Analogue Scale.

The study protocol is shown in Figure 1. Randomised allocation between groups A and B was performed using a random number table by a pharmacist who was not involved in the trial, at the pharmaceutical department of Kakamigahara Hospital. Participants were randomly assigned to group A or B in a double-blind design, and were administered sofpironium bromide gel or a placebo gel once a day, on the external skin, over the parotid and submandibular glands. Medical staff instructed participants on how to use topical gel on bilateral external skin over the parotid and submandibular glands, and ensured that they applied it correctly. Following the instruction, the patients themselves, their family members or medical staff applied the appropriate amount of gel. It does not result in differences in the dose of gel among patients. Group A was treated with sofpironium bromide gel for the first 6 weeks, followed by a 2-week washout period and then placebo gel administration for 6 weeks. In contrast, group B was treated with placebo gel for the first 6 weeks, followed by a 2-week washout period and then 6 weeks of sofpironium bromide gel administration. From the start of the administration period, both groups were observed every 2 weeks, for up to 16 weeks, which corresponded to 2 weeks after the end of the second 6-week application period (Fig. 1). To reduce any carry-over effects, we set the 2-week washout period.

Once every 2 weeks, we assessed the following four items, comprising a primary end-point and three secondary end-points: (a) 1-min saliva volume (g/min) (primary end-point), (b) objective assessments of salivation (severity (range 1–5) and frequency (range 1–4) on the DSFS^24,31^ and Nocturnal Hypersalivation Rating Scale (NHRS; range 0–4))^32^ and (c) a subjective assessment of salivation (Visual Analogue Scale (VAS; range 0–10).^33^ Furthermore, we confirmed subjective and objective side-effects based on medical interview and physical examinations.

For sample size calculation and statistical analyses, we used the statistical software EZR (Easy R) version 1.54 for Windows (Saitama Medical Center, Jichi Medical University, Saitama, Japan),^34^ which is a graphical user interface for R (The R Foundation for Statistical Computing, Vienna, Austria; https://cran.r-project.org/bin/windows/base/). Differences in continuous variables, such as age and age at onset, were analysed between groups A and B with an unpaired *t-*test. Differences in categorical variables, such as gender, were analysed with Fisher's exact test. To compare changes in 1-min saliva volume from the initiation of the study in groups A and B, a paired *t-*test was used. Furthermore, we directly compared changes in 1-min saliva volume between groups A and B with a unpaired *t-*test. To compare changes in objective and subjective salivation assessment scale scores from the beginning of the study in groups A and B, the Wilcoxon signed-rank test was used. A *P*-value of <0.05 was defined as significant for the current study.

## Results

**Fig. 2:**
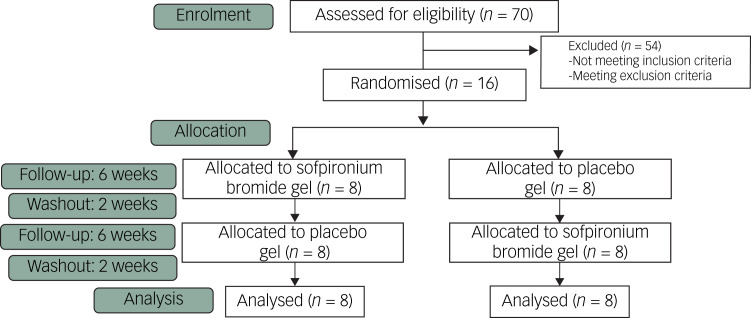
Consolidated Standards of Reporting Trials (CONSORT) flowchart for the study.

The Consolidated Standards of Reporting Trials (CONSORT) flowchart of this study is shown in Figure 2. Details on the participants who were eventually chosen are shown in Table 1. There were no significant differences in demographic variables between groups A and B (*P* > 0.05). No participants dropped out in either group; all participated to the end of the observation period. During the drug application period, three participants complained of mild itching at the site of application; however, there was no visible change in the skin surface, and the itching recovered spontaneously. One of the three participants reported the same issue during the placebo application. No other systemic or localised side-effects were noted.

**Table 1:** Demographic variables of participating patients with treatment-resistant schizophrenia: comparison between groups A and B

Patient number	Group	Gender	Age (years)	Age at onset (years)	Duration of illness (months)	PANSS total score	Chlorpromazine equivalent (mg/day) before clozapine	Clozapine dose (mg/day)	Duration of clozapine (months)	Salivation rate before clozapine (g/min)
1	A	Male	29	19	10	83	1960	600	29	1.58
2	A	Male	38	19	19	102	2400	600	13	1.45
3	A	Male	50	37	13	108	1200	300	68	3.24
4	A	Female	28	21	7	67	600	200	72	1.56
5	A	Female	33	15	18	47	1400	250	57	1.88
6	A	Female	47	19	28	78	685	200	60	1.76
7	A	Female	54	19	35	85	1509	300	96	1.43
8	A	Female	57	21	36	108	1000	600	104	1.89
Group A total		3 male, 5 female	42.0 ± 10.7	21.3 ± 6.2	20.8 ± 10.4	84.8 ± 19.9	1344.3 ± 575.7	381.3 ± 173.1	62.4 ± 28.7	1.85 ± 0.55
9	B	Male	28	15	13	112	800	400	85	1.37
10	B	Male	30	18	12	92	1103	300	20	1.93
11	B	Male	34	19	15	38	1400	250	5	2.24
12	B	Male	47	20	27	100	1260	500	104	2.67
13	B	Female	35	20	15	44	1400	300	37	1.44
14	B	Female	46	36	10	67	600	100	88	2.53
15	B	Female	48	14	34	97	600	600	132	2.98
16	B	Female	56	19	37	97	600	600	123	3.91
Group B total		4 male, 4 male	40.5 ± 9.4	20.1 ± 6.4	20.4 ± 10.0	80.9 ± 25.9	970.4 ± 337.5	381.3 ± 165.7	74.3 ± 44.8	2.86 ± 0.79

PANSS, Positive and Negative Syndrome Scale.

**Fig. 3:**
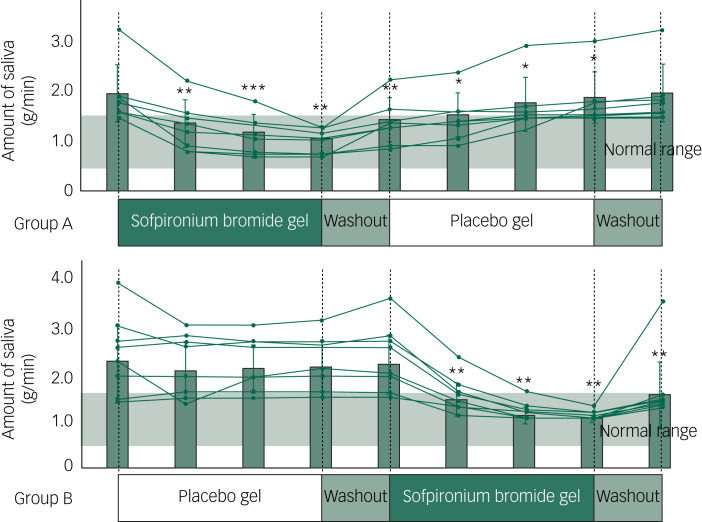
Group-wide changes in 1-min saliva volume in groups A and B. Bar graphs indicate changes in the mean ± s.d. of the 1-min saliva volume in groups A and B. The mean ± s.d. of the 1-min saliva volume for 16 age- and gender-matched healthy individuals (seven men and nine women) is presented as the normal range (0.944 ± 0.075). *P < 0.05, **P < 0.01, ***P < 0.001 (compared with the baseline).

Individual 1-min saliva volumes (primary end-point) (line graph) as well as the mean and s.d. (bar graph) for groups A and B as measured every 2 weeks are shown in Figure 3. Compared with the 1-min saliva volume in groups A and B at the beginning of the study, the saliva volume was significantly decreased (>30%) in group A during the second week of sofpironium bromide gel therapy (*P* = 0.0011). At the 4- and 6-week follow-ups, the saliva volume continued to decrease (*P* < 0.001). In contrast, the saliva volume increased after the 2-week washout period compared with the sixth week of sofpironium bromide gel therapy, but it was still significantly lower than the baseline volume (*P* = 0.0049). Therefore, it was assumed that the drug remained efficacious during that time. Subsequently, the drug's efficacy gradually decreased in the second week of the placebo gel application, indicating a return to baseline.

In group B, there was no significant decrease in saliva at the second or sixth week of placebo gel application, or during the 2-week washout period (Fig. 3). The slight decrease observed in the beginning gradually became less noticeable, albeit not significantly (*P* > 0.05), indicating the weakening of the placebo effect. As in group A, an approximately 30% reduction in saliva was confirmed at the second week of sofpironium bromide gel therapy (*P* = 0.0018). Moreover, the saliva volume decreased at the 4- and 6-week follow-ups, and the significant effect lasted 2 weeks after the washout period (*P* < 0.01).

**Fig. 4:**
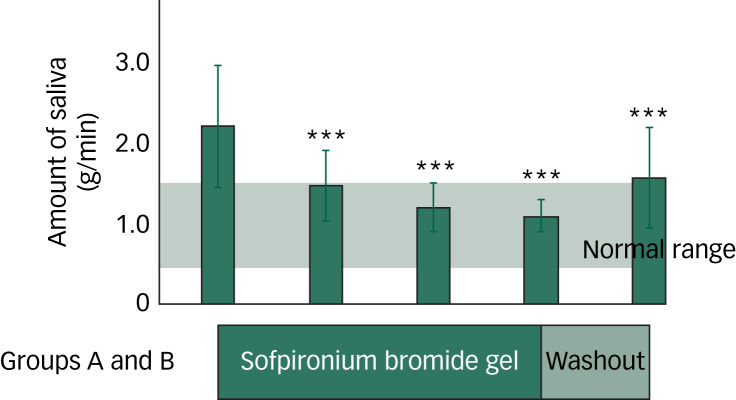
Changes in the 1-min saliva volume for all 16 participants (groups A and B combined). ***P < 0.001 (compared with the baseline).

When the sofpironium bromide gel therapy conditions in groups A and B were combined for analysis (Fig. 4), the changes in the 1-min saliva volume from the start of sofpironium bromide gel therapy grew larger (*P* < 0.001). Compared with the baseline, saliva volumes were decreased by 34%, 46% and 49% during the second, fourth and sixth weeks of sofpironium bromide gel treatments, respectively. A decrease of 29% was maintained in the second week of the washout period (*P* < 0.001). Furthermore, when the differences in the saliva volume between groups A and B were investigated every 2 weeks, the 1-min saliva volume significantly differed between groups A and B at the second, fourth, sixth, 12th and 14th week during sofpironium bromide gel therapy, as well as at the eighth week after the 2-week washout period (*P* < 0.05). In contrast, there were no significant changes in the saliva volume between groups A and B at the tenth and 16th week (*P* > 0.05), indicating a possible carry-over effect of at least 2 weeks during the first 2-week washout period only.

**Fig. 5:**
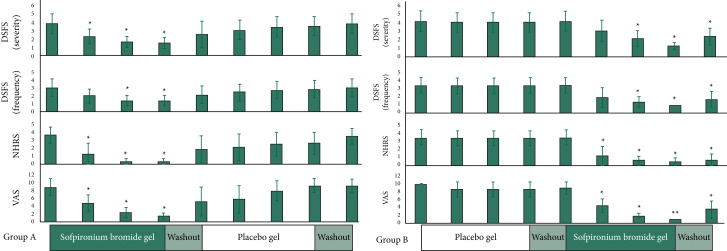
Changes in Drooling Severity and Frequency Scale (severity/frequency), Nocturnal Hypersalivation Rating Scale and visual analog scale scores in groups A and B. DSFS, Drooling Severity and Frequency Scale; NHRS, Nocturnal Hypersalivation Rating Scale; VAS, Visual Analogue Scale. *P < 0.05, **P < 0.01 (compared with the baseline).

Objective and subjective salivation assessment scale scores (the secondary end-points) every 2 weeks for groups A and B are shown in Figure 5. On all scales, group A scored significantly below baseline in the second, fourth and sixth weeks of sofpironium bromide gel administration (*P* < 0.05). After 8 weeks, scores gradually returned to baseline. In group B, the scores decreased from the tenth to the 14th week during sofpironium bromide gel administration (*P* < 0.05). The decrease remained significant even in the 16th week (*P* < 0.05), which occurred at the end of the 2-week washout period. These changes in the secondary end-points (Fig. 5) were similar to those in the primary end-point (Fig. 3).

These results showed a decrease in the primary end-point (1-min saliva volume), as well as improvements in the secondary end-points (objective and subjective scale scores). The effect was objectively and subjectively noticeable 2 weeks into sofpironium bromide gel therapy. The effect was maintained during sofpironium bromide gel therapy, but disappeared after the regimen was stopped. The mean ± s.d. of the VAS score for group A was 8.75 ± 2.17 at the initial assessment and 1.38 ± 0.70 at 6 weeks after sofpironium bromide gel administration. In group B, it was 10.00 ± 0 at the initial assessment and 1.00 ± 0 at 6 weeks after sofpironium bromide gel administration. In both groups, subjective symptoms improved dramatically. In group B, the VAS scores improved from 10 to 1 in all eight particpants.

To support our findings, a survey was conducted after the trial; the survey revealed that 14 out of the 16 (87.5%) participants wished to continue using sofpironium bromide gel, indicating that the effectiveness and tolerability of the treatment were high.

## Discussion

This is the first double-blind, controlled crossover study to confirm the efficacy of sofpironium bromide gel (5% ECCLOCK® gel) on clozapine-induced hypersalivation in patients with treatment-resistant schizophrenia. All 16 participants completed the trial without experiencing any significant side-effects. Our double-blind comparative study confirmed that applying sofpironium bromide gel once a day on the skin above the parotid and submandibular glands significantly reduces the saliva volume in patients with treatment-resistant schizophrenia who experience hypersalivation after receiving clozapine therapy. The effect became noticeable in the second week, with a 30% reduction in saliva volume. The effect persisted in the fourth and sixth weeks, with a 40% decrease. Even after sofpironium bromide gel was discontinued, the effect lasted at least 2 weeks. Furthermore, there were significant improvements in objective and subjective salivary symptoms. Fourteen out of 16 patients (87.5%) wished to continue with the treatment. Thus, tolerance was determined to be high.

We observed that the effects of sofpironium bromide gel on clozapine-induced hypersalivation appeared at the second week of administration, and salivation continued to decrease at the fourth and sixth weeks. Similar to our findings, a previous study in individuals with primary axillary hyperhidrosis reported that sweat volume decreased significantly at the second week of sofpironium bromide gel administration to the axilla, and continued to decrease at the fourth and sixth weeks.^23^ These findings suggest that sofpironium bromide gel could address hypersalivation and hyperhidrosis at the same rate via focal anticholinergic actions.

None of our patients experienced systemic side-effects after sofpironium bromide gel administration; all side-effects were localised, such as mild itching. These results were similar to those of a previous study in individuals with primary axillary hyperhidrosis.^23^ These findings suggest that sofpironium bromide gel may be better tolerated than oral administration or injection of anticholinergic drugs.

Even 2 weeks after the washout period, the effect of sofpironium bromide gel on hypersalivation was still demonstrable. Although we did not use a questionnaire to assess side-effects, we did not observe any systemic side-effects related to the sofpironium bromide gel, based on medical interview and physical examinations. Considering its continuous effect over at least 2 weeks, sofpironium bromide gel might not degraded quickly, but it also did not cause any severe side-effects.

Oral anticholinergic drugs can induce cognitive impairment as a side-effect. Although we did not assess adverse effects of cognitive impairment related to sofpironium bromide gel, a previous study has demonstrated that glycopyrrolate, a tolerable anticholinergic agent, was not associated with cognitive adverse events.^4^ Considering that sofpironium bromide is a derivative of glycopyrronium, the sofpironium bromide gel has potentially no such cognitive adverse effect. Although adverse events during the glycopyrrolate trial were mild and transient, adverse eventsrelated to the anticholinergic effects, such as orthostatic hypotension and palpitations, were observed.^4^ In contrast, there were no systemic or localised side-effects except for mild itching in this study. Therefore, we suggest that sofpironium bromide gel is a more toleratable anticholinergic agent than glycopyrrolate.

There are some limitations to the interpretations of our findings. First, our sample size (*N* = 16) was relatively small. Second, the study period was short (16 weeks). Third, although we assessed saliva volume per minute during rest and wakefulness, the saliva volume fluctuates throughout the day and is influenced by various conditions, such as sleep, diet and mental stress. Thus, the effect of sofpironium bromide gel on saliva volume throughout the day is unknown.

In conclusion, our findings showed that sofpironium bromide gel could treat hypersalivation, and thus may reduce the risk of aspiration pneumonia in patients with treatment-resistant schizophrenia who experienced hypersalivation after receiving clozapine therapy. Compared with conventional oral administration of anticholinergic drugs, sofpironium bromide gel has the advantage of reducing systemic side-effects, such as severe decreases in gastrointestinal motility, cognitive impairment and photophobia. Therefore, sofpironium bromide gel treatment would improve quality of life in patients. As sofpironium bromide gel application is anticipated in intractable neurological diseases that cause hypersalivation (e.g. ALS, Parkinson's disease and polio), further clinical trials on the clinical application of sofpironium bromide gel are warranted.

## Data Availability

Data are not publicly available due to their containing information that could compromise research participant privacy/consent.
